# Validation of a Zio XT Patch Accelerometer for the Objective Assessment of Physical Activity in the Atherosclerosis Risk in Communities (ARIC) Study

**DOI:** 10.3390/s24030761

**Published:** 2024-01-24

**Authors:** Anis Davoudi, Jacek K. Urbanek, Lacey Etzkorn, Romil Parikh, Elsayed Z. Soliman, Amal A. Wanigatunga, Kelley Pettee Gabriel, Josef Coresh, Jennifer A. Schrack, Lin Yee Chen

**Affiliations:** 1 Bloomberg School of Public Health, Johns Hopkins University, Baltimore, MD 21205, USA; letzkor1@jhmi.edu (L.E.); awaniga1@jhu.edu (A.A.W.); coresh@jhu.edu (J.C.); jschrac1@jhu.edu (J.A.S.); 2 Regeneron Pharmaceuticals Inc., Tarrytown, NY 10591, USA; jacek.urbanek@regeneron.com; 3 School of Public Health, University of Minnesota, Minneapolis, MN 55455, USA; parik075@umn.edu; 4 Section on Cardiovascular Medicine, Department of Medicine, Wake Forest School of Medicine, Winston-Salem, NC 27157, USA; esoliman@wakehealth.edu; 5 Center on Aging and Health, Johns Hopkins University, Baltimore, MD 21218, USA; 6 School of Public Health, University of Alabama at Birmingham, Birmingham, AL 35294, USA; gabrielk@uab.edu; 7 Medical School, University of Minnesota, Minneapolis, MN 55455, USA; chenx484@umn.edu

**Keywords:** Zio XT Patch, validation, wearables, ActiGraph GT3X

## Abstract

Background: Combination devices to monitor heart rate/rhythms and physical activity are becoming increasingly popular in research and clinical settings. The Zio XT Patch (iRhythm Technologies, San Francisco, CA, USA) is US Food and Drug Administration (FDA)-approved for monitoring heart rhythms, but the validity of its accelerometer for assessing physical activity is unknown. Objective: To validate the accelerometer in the Zio XT Patch for measuring physical activity against the widely-used ActiGraph GT3X. Methods: The Zio XT and ActiGraph wGT3X-BT (Actigraph, Pensacola, FL, USA) were worn simultaneously in two separately-funded ancillary studies to Visit 6 of the Atherosclerosis Risk in Communities (ARIC) Study (2016–2017). Zio XT was worn on the chest and ActiGraph was worn on the hip. Raw accelerometer data were summarized using mean absolute deviation (MAD) for six different epoch lengths (1-min, 5-min, 10-min, 30-min, 1-h, and 2-h). Participants who had ≥3 days of at least 10 h of valid data between 7 a.m–11 p.m were included. Agreement of epoch-level MAD between the two devices was evaluated using correlation and mean squared error (MSE). Results: Among 257 participants (average age: 78.5 ± 4.7 years; 59.1% female), there were strong correlations between MAD values from Zio XT and ActiGraph (average *r*: 1-min: 0.66, 5-min: 0.90, 10-min: 0.93, 30-min: 0.93, 1-h: 0.89, 2-h: 0.82), with relatively low error values (Average MSE × 10^6^: 1-min: 349.37 *g*, 5-min: 86.25 *g*, 10-min: 56.80 *g*, 30-min: 45.46 *g*, 1-h: 52.56 *g*, 2-h: 54.58 *g*). Conclusions: These findings suggest that Zio XT accelerometry is valid for measuring duration, frequency, and intensity of physical activity within time epochs of 5-min to 2-h.

## 1. Introduction

Physical activity (PA) is an important, modifiable risk factor for various diseases, chronic conditions, and functional outcomes [1]. In contrast to the traditional methods of assessing physical activity, that rely on self-reported and questionnaire-based data which lack granularity, objectivity, and accuracy, previous studies have demonstrated the reliability of accelerometers, such as ActiGraph, in capturing physical activity over extended periods with minimal user involvement [2,3,4,5]. Due to relatively low cost and ease of implementation, the use of accelerometers to directly estimate PA in research and clinical settings has increased exponentially over the past two decades [2]. These accelerometers use micro-electromechanical systems (MEMS) sensors to measure body acceleration (expressed in gravitational-units: *g*) in three orthogonal axes, with sampling frequencies usually ranging from 10 to 100 Hz (samples per second) per axis [6,7]. They are relatively small, wireless, and non-invasive, with a long battery life, generating a direct and continuous assessment of PA across multiple levels of exertion [7].

Daily PA is closely related to an individual’s cardiovascular health [8]. It has also been reported that heart rhythms are notably influenced by physical activity [9,10]. Knowledge of an individual’s posture and the intensity of physical activity may also impact the interpretation of heart rate readings [11,12]. Existing research indicates that certain symptoms, such as elevated heart rate during moderate or vigorous physical activity, have been observed. However, there is a gap in the literature regarding the integration of accelerometer sensors with ECG data to contextualize heart rate readings in relation to the level of co-occurring physical activity. Devices that simultaneously measure heart rhythms and PA in free-living settings allow researchers to study their associations, including the contribution of bouts of PA to heart rhythms [13], as well as to enhance the accuracy of interpreting heart rate patterns, aiding in the assessment of the necessity for further examination and interventions. In 2012, iRhythm introduced the Zio XT patch (Zio XT), a small, chest-worn ambulatory heart rhythm monitor equipped with a tri-axial MEMS accelerometer. Implementing simultaneous measurements of raw electrocardiogram (ECG) and accelerometry in one small, non-invasive device that can be worn on the body for multiple days in free-living settings provides the opportunity to characterize temporally-matched associations between free-living PA and heart rate as well as their joint associations with adverse health outcomes such as cardiovascular disease. However, the accelerometer used in the Zio XT device has different mechanical and processing specifications from other accelerometers used in biomedical research. Specifically, the frequency of observations collected by the MEMS accelerometer (1.56 observations per second (Hz) per axis) and the dynamic range are lower than other research grade devices.

Therefore, the objective of this analysis was to compare acceleration data collected using the ActiGraph wGT3X-BT (ActiGraph LLC, Pensacola, FL, USA) PA monitor worn on the hip with the chest-worn Zio XT monitor using mean absolute deviation (MAD [14,15,16]) for quantifying activity intensity extracted from raw accelerometer data. Data was obtained from participants who wore both devices simultaneously in the Atherosclerosis Risk in Communities (ARIC) Study Visit 6 (2016–2017). The ActiGraph is a research-grade accelerometer and US Food and Drug Administration (FDA)-approved medical device commonly used in scientific studies and was used as the reference measure for this study. We hypothesized that given the trunk location of both devices and the type and sampling parameters of the Zio XT accelerometer, the characteristics of activity intensity quantified using MAD from the Zio XT are comparable to the characteristics of activity intensity produced by the ActiGraph.

The work is organized as follows: Section 2 describes the dataset and the conducted experiments to evaluate the agreement between accelerometer data from Zio XT and ActiGraph, and Section 3 describes the results. Section 4 discusses the findings and possible future research directions; and finally, Section 5 summarizes the conclusions of the study.

## 2. Methods

*Participants*:

The ARIC study is a prospective, ongoing, community-based cohort study that began in 1987–1989 to investigate the etiology and clinical outcomes of atherosclerosis [17]. Adults aged 45–64 years were recruited from four field centers: Forsyth County, NC; Jackson, MS; Minneapolis suburbs, MN; and Washington County, MD. To date, ARIC has completed eight follow-up visits [18]. The ARIC study was approved by the Institutional Review Board at each participating study, and informed consent was obtained from all participants. Data for the current analysis were collected as part of ARIC visit 6 when all participants were asked to wear a Zio XT patch for up to two weeks. Participants were excluded if they had a skin allergy to patch adhesives or a history of cardiac electronic device implants. During the first 6 months of visit 6, a subset of participants were also offered to wear an ActiGraph accelerometer as part of a physical activity and falls study. More complete information and inclusion/exclusion criteria for the Zio XT data collection in ARIC visit 6 are presented elsewhere [19,20].

*Data Collection*:

**Zio XT**: The Zio XT device (iRhythm, San Francisco, CA, USA) is a small, FDA-approved, chest-worn device for ambulatory cardiac monitoring. It collects ECG data using two electrodes with a sampling frequency of 199.8 Hz [21]. It is attached to the left side of the upper chest area via a skin-safe adhesive. It is water-resistant and can be worn continuously for up to 14 days. After data collection, the device is sent to iRhythm where data are downloaded, a clinical report is produced, and raw data are generated. 

The Zio XT device also collects accelerometry data in three orthogonal axes (*x*, *y* and *z*). These data are sampled at a rate oSSSf about 1.56 observations per second (Hz) per axis. Accelerometry data are stored in binary “.zacl” files where each sample is represented as an 8-bit unsigned integer, ranging from 0 to 2^8^-1. The dynamic range of the accelerometer implemented in the Zio XT device is ±2 *g*. The data are structured in trifectas of *x*, *y*, and *z* where every third observation forms a time series of acceleration in their respective axis. Therefore, if *a* represents the vector of size *N* of all accelerometry observations then *x* = (*a*_1_ *a*_4_ *a*_7_ … *a*_N−2_), *y* = (*a*_2_ *a*_5_ *a*_8_ … *a*_N−1_) and, *z* = (*a*_3_ *a*_6_ *a*_9_ … *a*_N_). Additional information on the measurement, including the start and finish date and time, can be extracted from a separate header (‘hea’) text file. We used the ‘postuR’ R package [22] for preprocessing the native Zio XT accelerometer data. 

**ActiGraph**: The ActiGraph device is a small PA monitor that collects tri-axial accelerometry data with a configurable sampling frequency of up to 100 Hz per axis. It is water- and dust-resistant (IP27) and can be worn on the hip, wrist, or ankle for up to 25 days, depending on the data collection parameters. Collected data can be downloaded locally using the proprietary ActiLife software (https://actilife.theactigraph.com/support/software/actilife/) to a “.gt3x” file containing 12-bit binary values of accelerations. The dynamic range of the ActiGraph device is ±8 *g*. Using the ActiLife device, the measurement start date and time can be extracted and the data converted to comma-separated values (CSV) text format for further analysis.

As a part of the ARIC physical activity and falls study, a subset of 536 participants wore an ActiGraph device on an elasticized belt on their right hip. Participants were instructed to wear the activity monitor during all waking h and to remove it for sleeping, bathing, and swimming for the following seven consecutive days. Participants returned the devices via US mail in a pre-addressed envelope. The ActiGraph devices were set to collect raw tri-axial accelerometry data with a sampling frequency of 40 Hz.

*Validation*:

For each participant, raw, tri-dimensional accelerometer data expressed in *g*-units were downloaded from both the Zio XT and ActiGraph devices and collapsed into one-dimensional vector magnitudes expressed as per Equation (1):

(1) $$r\left(t\right)=\sqrt{x^{2}\left(t\right)+y^{2}\left(t\right){+z}^{2}\left(t\right)},\;$$

where *t* represents the time and *x*, *y*, and *z* represent the data collected on three orthogonal axes. Next, the mean absolute deviation [23] of the vector magnitude (MAD) values over predetermined time windows were calculated as per Equation (2):

(2) $$MAD\left(t,\;H\right)=\frac{1}{H}\sum_{h=0}^{H-1}\left|r\left(t+h\right)-\bar{r}\left(t;H\right)\right|,$$

where *H* corresponds to the number of samples in each time interval, *h* corresponds to each sample in the time interval, and $\bar{r\;}\left(t;H\right)=\frac{1}{H}\sum_{\;h=0}^{\;H-1}\;r(t+h)$. We used the following time windows to calculate the MAD values for the two devices and examine their correlation: one minute, five minutes, ten minutes, thirty minutes, one hour, and two hours.

For each participant, the calculated MAD time series data were time-synchronized between the Zio XT and ActiGraph devices, using the timestamp variable of the respective monitors. Non-wear periods in the ActiGraph device were defined as 90-min intervals of consecutive one-min activity count values equal to zero (obtained using R package ‘arctools’ [24,25]). Non-wear periods in the Zio XT device were determined based on the non-wear periods detected in the ECG data. To that end, the ECG time series was smoothed using a moving average with a 2-s rectangular window and timepoints where the difference between the original and smoothed ECG data was smaller than 0.1 mV were labeled as non-wear. Non-wear periods from each device were not taken into account when calculating the MAD. We included participants who had at least three days with at least ten h of overlapping device wear-time between 7 a.m and 11 p.m. The accelerometer data from both devices were also visually examined for potential device error during data collection.

For each time window duration, we calculated the participant-specific Pearson’s correlation coefficients and mean squared error (MSE) values between the Zio XT and ActiGraph MAD values. The comparison analyses were averaged across participants separately for each of the six time windows: 1-min, 5-min, 10-min, 30-min, 1-h, and 2-h time windows. Bland–Altman plots were used to assess systematic differences or possible outliers between the measurements of the two devices. All analyses were performed using R version 4.2.0 and the R packages ‘arctools’ [24] for ActiGraph non-wear detection, ‘Metrics’ [26] for calculation of the MSE, ‘lsr’ [27] for calculation of the MAD, and ‘postuR’ [22] for preprocessing the Zio accelerometer data. 

## 3. Results

**Figure 1:**
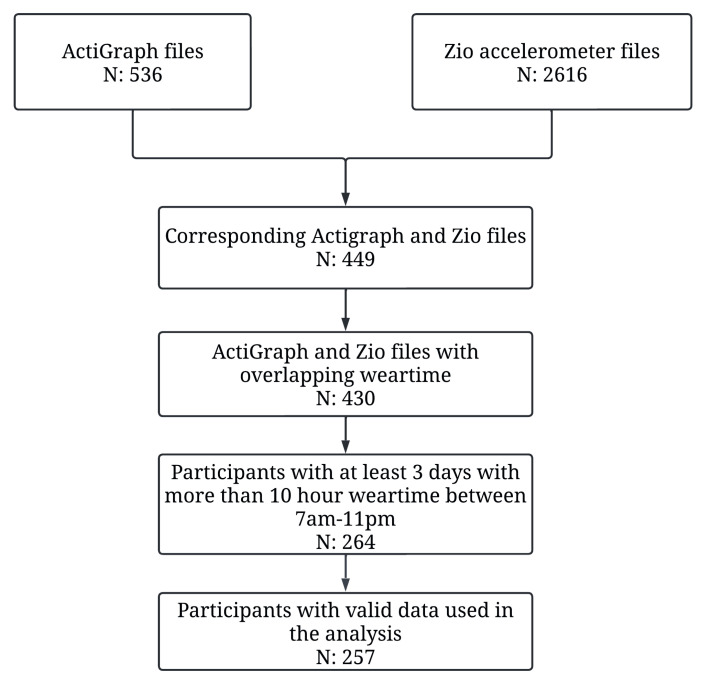
Flow diagram of the cohort selection process.

*Participants*:

Our final dataset included data from 257 participants (average age: 78.48 (standard deviation (SD) = 4.65) years, 59.14% female, and 83.27% white) (Figure 1, Table 1). The accelerometry data included an average of 4.69 (sd = 1.09, min = 3, max = 7) days per participant.

**Figure 2:**
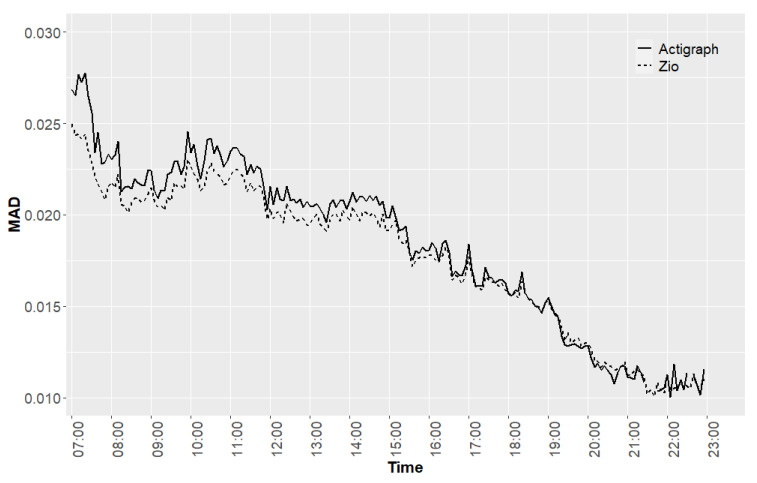
Average ActiGraph and Zio XT MAD values for 5-min windows, averaged across all valid days across all participants.

*Validation*:

We calculated and compared the correlation and MSE values for the recordings of the two accelerometers for the six time windows. Figure 2 shows the diurnal patterns of the MAD values using a 5-min window length for both the ActiGraph and Zio XT devices, averaged across all participants and days, with a Pearson correlation coefficient of 0.995 and an MSE of 9.42 × 10^−7^ *g*^2^. Visually, the difference between the average MAD values calculated for the 5-min windows seemed to be smaller during times of lower activity (afternoon/evening). 

**Figure 3:**
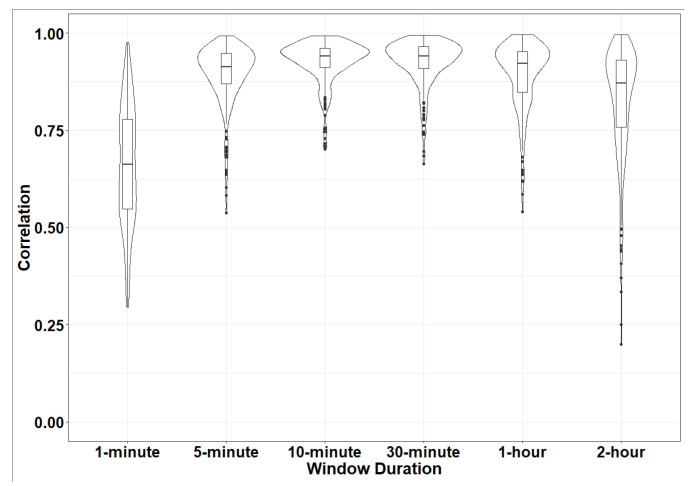
Distribution of participant-level mean correlation values between the acceleration recordings for ActiGraph and Zio XT devices for each participant, averaged for each participant for the respective time window size.

**Figure 4:**
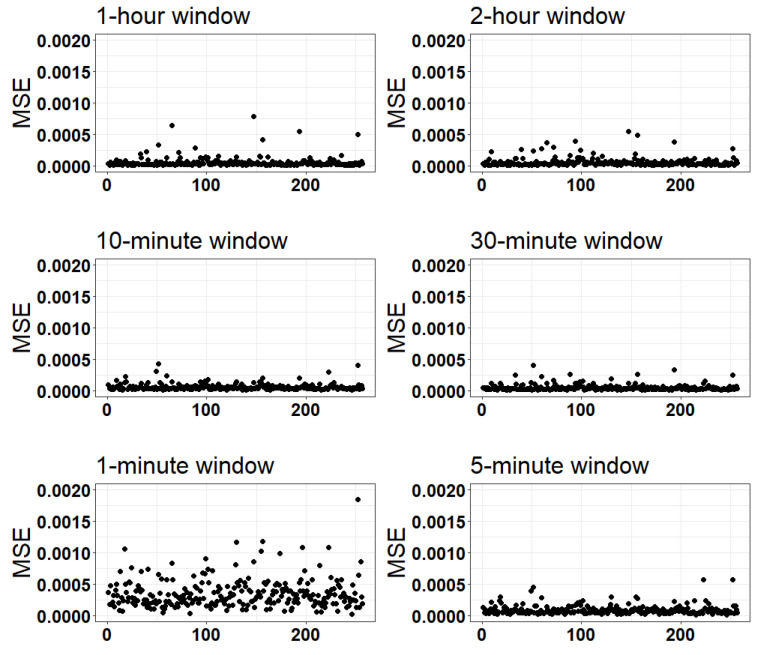
Mean squared error (MSE) values for each window length. Figures show the mean MSE value for each participant for all periods of the respective window size.

Table 2 and Figure 3 show the distribution of the average correlation values for each participant across each time window. The median of the average correlation value for each participant ranged from 0.94 for the 10-min and 30-min windows to 0.66 for the 1-min time window. The results showed a high correlation (>0.8) among observations for most participants. For example, in the 5-min time window, 21 participants (8.17%) had a correlation less than 0.8 (Table 2). Table 3 and Figure 4 show the distribution of the average MSE values for each participant for each time window. The metrics in 1-min and 5-min windows tended to have higher MSE values, while the MSEs values were lower for the longer window durations. For example, by increasing the MAD calculation window length, both the average and median MSE error decreased from 349.37 × 10^−6^ *g*^2^ and 295.42 × 10^−6^ *g*^2^ for the 1-min window length to 45.46 × 10^−6^ *g*^2^ and 29.91 × 10^−6^ *g*^2^ for the 30-min window length, respectively.

**Figure 5:**
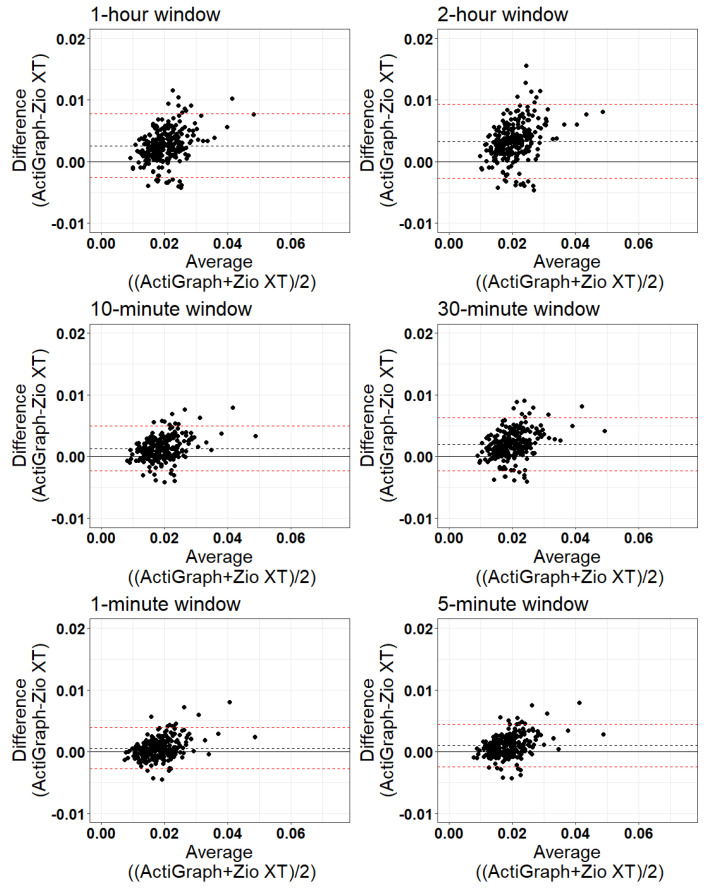
Bland–Altman plots for each window length. Figures show the average MAD value for each participant for all periods of the respective window size. The solid black line indicates the mean difference. The dash red lines indicate the lower and upper 95% limits of agreement.

**Figure 6:**
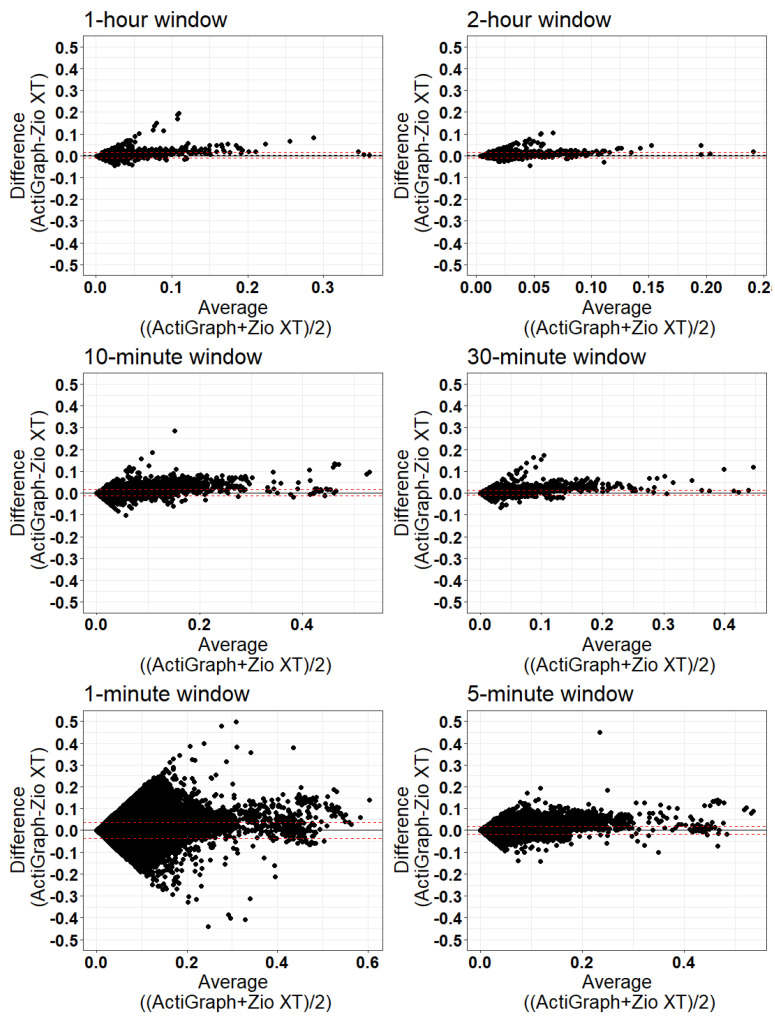
Bland–Altman plots for each window length. Figures show all the MAD values for all participants and all periods of the respective window size. The solid black line indicates the mean difference. The dash red lines indicate the lower and upper 95% limits of agreement.

Bland–Altman plots comparing the MAD values from the Zio XT to ActiGraph (reference) devices for the data combined across participants as well as the individual MAD values are presented for each time window in Figure 5 and Figure 6, respectively. Table 4 and Table 5 summarize the bias (mean difference) and limits of agreement (calculated as the mean difference ± 1.96 × standard deviation) and the percentage of samples outside the limits of agreement for each time window for MAD calculation for each participant overall (Figure 5) and for each individual point (Figure 6), respectively. In both comparisons of the MAD values from the ActiGraph and Zio XT devices for each participant separately and for each sample point separately, less than 10% of the differences were outside the limits of agreement (5.06–8.17% for the differences averaged for each participant, and 3.24–5.74% for the differences for individual sample points). 

## 4. Discussion

We compared the PA measurement of tri-axial accelerometry data collected using ActiGraph and Zio XT devices in a subsample of ARIC participants. The results indicated very high population- and participant-level correlations between the two devices, with an average of the correlation coefficient and median of the correlation coefficient of 0.66–0.93 and 0.66–0.94, respectively. Our results suggest that with the increasing window length, the correlation between the acceleration MAD values from the two devices increases, and the difference (MSE) between the two decreases, with the 5-min epochs already showing a high correlation (0.90). However, this improving trend attenuates at time windows longer than 1 h. The improvement in the correlation between the data from the two accelerometers is expected, as with longer window durations, the average values of the MAD are smoother and the effects of de-synchronization across the device clocks are reduced. Collectively, these results suggest that it is feasible to use the accelerometer in the Zio XT device to measure PA in population and clinical research within medium-to-long time epochs (e.g., 5 min to 1 h). 

There are several potential reasons for the differences between the MAD values calculated from the accelerometer data of the two devices. The measurement parameters of the sampling frequency and the resolution of quantization of the analog acceleration signal differ across the two devices. The sampling frequency (*fs*) is associated with an anti-aliasfiltering [28] that removes all frequency components above *fs*/2 (Nyquist frequency), resulting in a reduction in the overall amplitude of the time signal (An anti-alias filter is a filter applied before sampling the signal to restrict the bandwidth of the signal to satisfy the Nyquist–Shannon sampling theorem. In short, this theorem states that the sampling rate must be greater than twice the highest frequency in the analog signal for a perfect reconstruction from the sampled data). While the precise parameters of signal conditioning in the Zio XT device are unknown to us, it is possible that they are different to those in the ActiGraph device. Previous research has shown that different accelerometer sampling frequencies can result in differences in measurements of movement such as activity counts [3]. However, in our findings, the large difference in the sampling frequency between the two accelerometers (1.56 Hz for the Zio XT device and 40 Hz for the ActiGraph device) did not prevent high correlation in the calculated MAD metric in time intervals longer than 1 min, suggesting that aggregated summaries from raw acceleration data would be similar between the two devices.

Due to the different body locations of the devices (hip vs. chest), accelerometers pick up components of human movement in different proportions. For example, the hip-worn ActiGraph device is more sensitive to hip rotation during ambulation, whereas the chest-worn Zio XT device may register residual arm movements and breathing patterns. This intuition can be viewed as analogical to the more general, well-known problem of contrasting accelerometry data collected by devices worn on different body locations. For instance, popular wrist-worn monitors [2,29,30,31] provide a good proxy of PA, as do the widely used hip- [32] or trunk-worn [33] devices. However, they fundamentally measure different types of movement and thus provide metrics on a different scale and may not be directly comparable. During highly active periods in the morning and early afternoon [20], variations in acceleration levels, including rapid and vigorous movements, may result in a larger difference between two devices’ data due to differences in the sensitivity and wear location when capturing high-intensity activities. In summary, some differences between the MAD values of the two devices in our study may be expected and do not affect the utility of the Zio XT device as a valid PA monitor. In addition, the different resolution of quantization introduces different levels of quantization noise to the digitized data. The ActiGraph device stores its data using 12-bit quantization, thereby representing acceleration values in 2^12^ = 4096 discrete bins in the range of ±8 *g* resulting in the resolution of 2 × 8 ÷ 4096 ≈ 0.004 *g*. The Zio XT device stores the data with 8-bit coding resulting in 2^8^ = 256 discrete levels. The dynamic range of the Zio XT accelerometer is ±2 *g* which leads to a resolution of 2 × 2 ÷ 256 ≈ 0.015 *g*. In theory, quantization noise is a random, zero-mean, uniformly distributed component of the signal. However, since we do not know the exact parameters of the data acquisition in both devices, the contribution of the quantization resolution on the compared metrics of PA cannot be ruled out. 

Although the Zio XT accelerometer compared favorably with the ActiGraph device, its low sampling frequency may be a limiting factor in incorporating its accelerometry data in certain types of research, particularly for finer grained analyses on raw data (for example, walking detection). However, the Zio XT device tends to be utilized mostly in clinical and epidemiologic research populations, which commonly use min-level or longer epochs for analysis. To this end, the accelerometry data collected by the Zio XT device appear to be a valuable and useful source of information on the general characteristics of free-living PA [16,34]. Moreover, use of the Zio XT technology for measuring PA presents an opportunity for the joint modeling of ambulatory ECG and free-living accelerometry, which could provide multiple novel research opportunities, including using heart rate to define accelerometry-based individual levels of exertion [13], observing dynamic cardiovascular characteristics during recovery after periods of moderate-to-vigorous PA, and defining the temporality and dynamic prediction of adverse events like atrial fibrillation or other types of arrhythmia. Even though research utilizing joint cardiovascular characteristics and PA has been conducted previously [35,36,37], the Zio XT device allows for observing these relationships on a much finer scale due to its high quality of raw ECG data and perfect time synchronization with within-device accelerometry. An important advantage of PA monitoring with the Zio XT device is reducing the costs and patient burden associated with the implementation of a separate accelerometry measurement protocol. 

Future research in this area should further validate aggregate summary variables of raw accelerometry data, including total and total–log volumes [38] and activity fragmentation [39] against characteristics widely associated with PA including age, mobility, physical performance, and fitness. 

With the increasing use of wearable sensors and home-based cardiac rehabilitation to improve participation rates, sensors that capture both ECG data and physical activity levels will be needed [40]. The Zio XT device offers continuous ECG monitoring and integration with physical activity data and flexibility for remote monitoring, making it a potentially valuable tool in cardiac rehabilitation. Integrating data on heart rhythms with information on activity levels provides a comprehensive understanding of the potentially bidirectional relationship between physical activity and cardiac health. This integrated data can guide the development of exercise prescriptions and rehabilitation plans tailored to an individual’s capabilities and needs and contribute to a more personalized and effective approach to cardiac rehab programs.

The strengths of this study include a large, well characterized study population with a high wear compliance for multiple days in the free-living environment. Our approach has several limitations as well: (1) The device protocols for the ActiGraph and Zio XT devices differed, which in turn imposed extensive pre-processing procedures. In addition, the ActiGraph data had multiple, often extensive, periods of non-wear time recorded as a result of the waking-h study protocol. Indeed, it is standard to ask participants to remove the hip-worn device for sleep, as it may often cause discomfort. (2) The use of quartz-based clocks in a vast majority of modern electronic devices, which suffer from random drift [41]. In research studies implementing PA measurement, this drift is often small and can be safely ignored. It becomes problematic mainly when synchronizing across multiple devices. Additionally, the ActiGraph and Zio XT devices were initialized using different desktop PCs, and the system times between these computers may not have been perfectly synchronous. As we used longer intervals for calculating the MAD, the errors caused by desynchronization were minimized, yet some minor differences could potentially remain. (3) We limited the scope of the presented analysis to the MAD metric in epochs of 1 min or longer. The MAD can be easily applied to accelerometry data regardless of the type and model of the device and has been shown to have strong performance in separating physical activity categories [14,23]. Future research needs to evaluate the agreement between other metrics of physical activity as measured by the Zio XT and ActiGraph devices. (4) The different body locations used in the data collection as a result of the study protocol and the specific characteristics of the two devices limit the comparability of the data collected from them. While our findings showed a strong correlation between the data collected from the chest-worn Zio XT device and the hip-worn ActiGraph device, the different placement locations of the accelerometers may have contributed to the differences in the recorded acceleration values and consequently to the calculated PA metric values. (5) The findings from this study may not translate directly to other populations such as pediatric populations. Future research needs to evaluate the agreement between physical activity as measured by the Zio XT and ActiGraph devices in other populations.

## 5. Conclusions

Our findings demonstrated validity between the MAD values calculated for the Zio XT devices and the MAD values calculated for the ActiGraph device in window lengths ranging from five minute to two hours. Overall, our results suggest that the Zio XT device appears to be an acceptable alternative to the ActiGraph accelerometer for measuring PA in a free-living environment that can be reliably used for the concurrent measurement of PA and heart rate rhythms. Additional work is warranted to determine the relevant cut points for determining the types and intensity of any physical activity.

## Figures and Tables

**Table 1:** Demographic characteristics of the participants (total N = 257).

Variable	Distribution
**Age, Mean (SD)**	78.48 (4.65)
**Gender: Female, N (%)**	152 (59.14%)
**Race: White, N (%)**	214 (83.27%)

**Table 2:** Summary of correlation between the Zio XT accelerometer data and ActiGraph accelerometer data (total N for each window = 257).

Window Length	Range of Correlation Coefficients *	Mean (SD)	Median (IQR)	N Subjects (%) with Correlation < 0.8
**1 min**	[0.30, 0.98]	0.66 (0.15)	0.66 (0.55, 0.78)	205 (79.77)
**5 min**	[0.54, 0.99]	0.90 (0.08)	0.91 (0.87, 0.95)	21 (8.17)
**10 min**	[0.70, 0.99]	0.93 (0.05)	0.94 (0.91, 0.96)	10 (3.89)
**30 min**	[0.66, 0.99]	0.93 (0.06)	0.94 (0.91, 0.97)	14 (5.45)
**1 h**	[0.54, 1]	0.89 (0.09)	0.92 (0.85, 0.95)	33 (12.84)
**2 h**	[0.20, 1]	0.82 (0.15)	0.87 (0.76, 0.93)	88 (34.24)

* Pearson’s correlation. *SD: standard deviation. IQR: interquartile range*.

**Table 3:** Summary of mean squared error (MSE) × 10^−6^ between the Zio XT accelerometer data and ActiGraph accelerometer data (total N for each window = 257).

Window Length	Range	Mean (SD)	Median (IQR)
**1 min**	[24.26, 1844.07]	349.37 (233.22)	295.42 (198.74, 440.73)
**5 min**	[11.68, 573.60]	86.25 (76.08)	62.99 (42.20, 100.68)
**10 min**	[8.20, 430.22]	56.80 (54.02)	37.82 (27.98, 66.92)
**30 min**	[4.71, 402.89]	45.46 (50.42)	29.91 (19.54, 49.06)
**1 h**	[4.96, 782.90]	52.56 (87.37)	29.12 (17.98, 48.70)
**2 h**	[3.96, 547.28]	54.58 (72.41)	31.66 (18.95, 58.59)

**SD: standard deviation.** *IQR: interquartile range*.

**Table 4:** Bland-Altman results for comparing calculated MAD values calculated for each window averaged for each participant for ActiGraph and Zio XT (Total N for each window = 257).

Window Length	Mean ActiGraph	Mean Zio	Bias (Mean Difference)	Limits of Agreement	% Outside Limits of Agreement
**1 min**	0.018	0.018	0.000575	(−0.0027, 0.0039)	5.06
**5 min**	0.019	0.018	0.000994	(−0.0024, 0.0044)	7.00
**10 min**	0.020	0.018	0.001312	(−0.0023, 0.0050)	7.00
**30 min**	0.021	0.019	0.002010	(−0.0023, 0.0063)	6.61
**1 h**	0.022	0.019	0.002583	(−0.0026, 0.0077)	8.17
**2 h**	0.022	0.019	0.003308	(−0.0027, 0.0093)	7.00

**Table 5:** Bland–Altman results for comparing all calculated MAD values for ActiGraph and Zio XT devices.

Window Length	N	Mean ActiGraph	Mean Zio	Bias (Mean Difference)	Limits of Agreement	% Outside Limits of Agreement
**1 min**	991,340	0.019	0.018	0.000600	(−0.0360, 0.0372)	5.74
**5 min**	198,161	0.019	0.018	0.001013	(−0.0169, 0.0189)	5.03
**10 min**	99,012	0.020	0.019	0.001328	(−0.0130, 0.01570)	4.33
**30 min**	32,917	0.021	0.019	0.002029	(−0.0105, 0.0146)	3.81
**1 h**	16,398	0.022	0.019	0.002600	(−0.0107, 0.0159)	3.24
**2 h**	8133	0.022	0.019	0.003321	(−0.0095, 0.0162)	4.06

## Data Availability

Anonymized data from the ARIC study are available through the National Heart, Lung, and Blood Institute Biologic Specimen and Data Repository Information Coordinating Center and can be accessed through the website (https://biolincc.nhlbi.nih.gov/studies/aric/). Interested researchers may additionally contact the ARIC study Coordinating Center to access the study data.
